# Sources and Resources‘The NHS … Should not be Condemned to the History Books’: Public Engagement as a Method in Social Histories of Medicine

**DOI:** 10.1093/shm/hkaa041

**Published:** 2020-05-15

**Authors:** Jennifer Crane

**Keywords:** public engagement, National Health Service, activism, childhood, methods

## Abstract

This article explores the public engagement work of the Cultural History of the National Health Service (NHS) project, conducted at the University of Warwick between 2016 and 2019 and aiming to explore the meanings attached to Britain’s NHS over its 70-year history. The article situates public engagement as a critical methodology for social historians of medicine, exploring how events deepened this project’s understandings of post-war welfare, childhood treatments and activist cultures. Through reflection on these themes, the article emphasises that public engagement can generate rich new forms of qualitative testimony, complementing archival documents; point us towards ‘hidden archives’; and challenge cultural visions of historical research as ‘condemning’ or ‘celebrating’ its subjects. Finally, the article provides critical reflection on the challenges of such work and argues that engagement around health makes visible the broader research challenges of emotional intensity, personal and professional boundaries, and the hierarchies ingrained in academic research.

At a museum event in St Fagans Museum, Wales, in June 2017, a team of historians left out nurses’ uniforms for visitors to sketch on. While initially reverentially reluctant to mark these sacred, but highly cleanable by design, fabrics, as the day continued, visitors wrote a variety of messages. Many related to policy: ‘No Paycap’, ‘Divided we fall and United we stand’, and ‘Keep it FREE’. Another message contended that this was ‘not a proper uniform’, but in fact overalls—as ‘real’ nurses wore caps and capes. Others drew equipment or added a pin badge. One of the most popular messages left was at the heart of this article, asserting that: ‘The NHS [is] a wonderful Welsh invention that should not be condemned to the history books! (emphasis is in original)’ Adding to this, other visitors wrote ‘I agree’, ‘Me too!’, and, even, ‘WE ALL AGREE!’. Survey material from the day further emphasised a sense of anxiety and concern about the role of history in relation to the National Health Service (NHS), and another visitor stated that: ‘I fear that soon the NHS will simply be another historical chapter. I am determined to fight this at every stage!’

These statements were provocative. In part, they suggest fears that the NHS may one day soon cease to exist and thus become ‘historical’. However, these statements may also prompt reflection on the relationships between history, historians and publics. What are the visions of history, which are revealed by engaged research? Does historical writing itself act to ‘condemn’ or ‘celebrate’ the NHS? What new sources and ideas can public engagement generate for historians and publics, and what are its challenges and limitations? This article looks to approach these questions, drawing on learning and examples from the Cultural History of the NHS project, a 5-year project at the University of Warwick, funded by the Wellcome Trust, with public engagement at its heart. The article aims to add to critical analysis of public engagement as a method in social histories of medicine, and to highlight its ability to nuance cultural understandings and public debate, and also generatively to create and collect new sources. To support this analysis, the article aims also to explain how engagement enriched the Cultural History of the NHS team’s understandings of the NHS; a key institution that, since its foundation in 1948, has shaped all British post-war histories of medicine, with publics affected as patients, workers, volunteers, supporters or critics. In particular, the article contends that engaged research brought ‘experience’ to the fore, enriching our work and enabling us to reach beyond the politics of experience towards a grasp of meanings and feelings.

The arguments and analysis of this article are throughout indebted to the work of social histories, oral histories, feminist histories, co-produced histories, histories from below and people’s histories, all of which have called not only for us to write histories which survey ‘everyday’ or ‘ordinary’ lives but which also harness public expertise into their very methods.^1^ Intersecting disciplines have also made relevant shifts since the 1980s: science and technology studies has sought to understand and nuance thinking around ‘expertise’ and social hierarchies; engagement and ‘entanglement’ have become central to the critical medical humanities; studies in medicine, philosophy, English and sociology have harnessed personal experience in their research.^2^ Throughout, this article cites and makes particular use of oral history and feminist research traditions, both of which provide critical models in terms of reflecting on, and looking to make visible, the conditions under which collaborative work was and is produced. In doing so, this article aims to further bolster and contribute to honest reflection about the challenges of such work for academics and to highlight the reliance of this work on significant personal effort and dedication.

While drawing on long-standing traditions, this article also looks to explore the distinct ways in which ‘public engagement’, specifically, may conceptualise and change relationships between historians and publics. Public engagement is becoming increasingly central to our field. Key funding agencies in the medical humanities, as well as the Research Excellence Framework, incentivise and encourage ‘engagement’ and ‘impact’.^3^ Multiple recent projects in the social history of medicine put engagement and experience at their core in exciting ways.^4^ Historians of medicine who collaborate with medical practitioners are influenced by the distinct but relevant practices of ‘public–patient involvement’, which are encouraged by health funders, as well as in requests from key journals to detail how patients and publics have been involved in published research.^5^ Thus, these distinct practical and theoretical contexts around ‘public engagement’ influence what social historians of medicine read, how we are funded, how we shape our results and the data that we generate.

In the last few years, articles and new journals have begun to discuss the meanings and logistics of events held in the names of ‘public engagement’ or ‘impact’ in very useful ways.^6^ This article contributes to this growing field. First, it outlines the scope of the Cultural History of the NHS project at the University of Warwick and its engagement events and digital presence. Secondly, it discusses ideas of engaged history as ‘celebrating’ or ‘condemning’ the NHS. The article describes how the 70th Anniversary of the NHS in 2018 provided new opportunities for our staff. These opportunities were primarily to *disseminate* our research, but the article argues that it was also possible to use such moments to amplify new cultural narratives and to present critical interventions. Thirdly, the article analyses how this project sought to construct engaged research collaboratively, arguing that this approach meant that the project had public benefits, as well as enriching research into childhood ‘experience’ by accessing adult memories and child drawings. Fourthly, the article outlines challenges involved in this work, notably a lack of public interest, disagreement and emotional fatigue. Overall, it suggests that project research—notably around activism—was strengthened by entering engagement events as open participants, acknowledging our multiple positions as historians, members of the public and patients. To conclude, the article argues that public engagement is a valuable method for social histories of medicine and more broadly a tool to assess our role as historians, our participation in social life and academic focus on ‘evaluation’ and ‘outputs’.

## The Project

The Cultural History of the NHS project at the University of Warwick was designed by Professors Roberta Bivins and Mathew Thomson in 2014. Bivins and Thomson argued that, despite the rich nature of political histories around the health service, there was yet ‘no history that addresses the realm of meaning, feelings, and representation, and none that responds to Nigel Lawson’s striking observation that “the National Health Service is the closest thing the English have to a religion”’.^7^ The project was interested in tracing how members of the public felt about the NHS, and how this had varied across demographic groups, time and space, as represented in culture and everyday life.

Engagement was central to this project from its outset, and the initial grant application, for a Wellcome Trust Senior Investigator Award, stated that engagement would not only be at the ‘core’ of this project, ‘to extend the dissemination of our findings’, but also, crucially, ‘an important research tool for evidencing and understanding popular attitudes and feelings.’ Further evidencing this commitment, in January 2016, the project appointed three Research Fellows and two Public Engagement Research Fellows, all of whom would be involved in producing their own engaged research.^8^

The project took a flexible approach to ‘public engagement’ and to the broad mandate of looking to understand public attitudes over time. For the first half of 2016, the project ran a pilot, looking to organise as many engagement activities as possible in the local area to scope out what was effective and what was not. Project staff visited local history societies, hospitals, campaign groups and, meeting a public request for a visit, a ladies’ choir. These initial visits began to expose the potential richness of this public work. At the visit to Leamington Spa History Society, in March 2016, for example, with little introduction, we quickly heard memories of vaccinations, polio and of children being told, before the introduction of the NHS, that they had better be ‘really sick’ if the doctor was to be called out. We heard distressing stories of poor quality hospital food, unavailable parking and poor cleanliness, but also positive tales of successful liver transplantations, childbirth and kind doctors.^9^

Following these scoping visits, and recognising a level of public enthusiasm for sharing such stories, we developed a model of Roadshows, loosely based on the Antiques Roadshow, where members of the public could bring along items, and see the items which we displayed, together to discuss material, emotional and everyday cultures of the NHS. We ran Roadshows with: the Thackray Museum, Leeds; the People’s History Museum, Manchester; Think-Tank Science, Birmingham; Rugby Art Gallery; Edinburgh Library; St Fagans Museums; and at the UNISON Health Conference, Liverpool. Often, because we then had multiple members of staff in these locations, we held evening workshops or panel sessions following the Roadshows, discussing specific issues that we were analysing in that moment. Our event in Manchester, for instance, was followed by a panel looking at how to trace activism around the NHS, which enabled us to discuss our current research questions and to meet members of local activist groups and NHS services.

These events were sometimes organised as we applied to present at specific venues and sometimes as specific venues approached us. Indeed, the whole public engagement strategy of this project was both responsive and proactive. We organised a ‘big draw’ session at University Hospitals Coventry and Warwickshire and curated art exhibitions there, both in response to our own interest in thinking about patient experience, but also reflecting the specific needs and preferences of the hospital staff. Alongside these physical forms of engagement, we also ran a website called peopleshistorynhs.org. The choice of title ‘People’s History’ was a difficult one, intended to appeal to a popular audience and to reflect this area’s interest in ‘challenging, widening and expanding the discipline as a whole’—yet it is also a title entwined with histories of labour and the left, which may have dissuaded certain audiences from engaging with our site.^10^ Nonetheless, the website went live in February 2016 and, in 1 year, had had 143,763 visits from 32,762 visitors. From February 2016 until March 2019, the website had 840,262 visits from 267,126 distinct visitors. These figures suggested that the website was gathering repeat visits and potentially also being used as a teaching, learning and reference tool. This sense was further validated by colleagues using the website in their teaching.^11^

Over this period, the website gained over 700 ‘members’, who had signed up to be able to share their memories with us and to read those of others. We received 133 memories directly shared through the ‘Share Your Stories’ section of the webpage and, additionally, over 300 comments on blog posts and encyclopaedia entries. Memories varied in length from 10 to 20 words to over 3,000 words, averaging at 200–300 words. We were also sent 32 photographs of artefacts, people and events, showing a material culture of the NHS, which may otherwise have been hidden in people’s homes.^12^ Looking to broaden out our online reach, in November 2016, we held an ‘Editathon’ with the Wikimedian-in-Residence at the Wellcome Library, Dr Alice White, adding information to relevant Wikipedia pages.

By taking a proactive and reactive approach, the project sought to avoid, as Stephen Hinchliffe *et al.* describe, a public health approach where ‘*the* public is understood to be already out there, a population waiting (passively) to be informed or incentivised about an issue’.^13^ Rather, we looked to access and enable dynamic participation from a range of publics, whether responding as individuals, members of communities, patients, friends, supporters or critics of the NHS and history writing. This approach, as Hinchliffe *et al.* describe, intends to be one which ‘incorporates multiple voices and recognises multiple forms of knowledge and experiences’.^14^

The Cultural History of the NHS project, then, was immensely privileged in a variety of ways. It benefited from the work of a considerable number of staff, generous funding and the engagement expertise forged at the Centre for the History of Medicine, University of Warwick, over several years. The size and reach of this project meant that we encountered multiple visions of engaged research—as condemnation, celebration and collaboration—but also that the challenges which we encountered were significant and revealed the inherent challenges of using genuinely engaged research as a method, even with high levels of resources and good will. Public engagement can be a valuable source for provoking historical thinking, but it is not generated simplistically; it must be planned, built, curated and analysed.

## Celebration

The first key vision of engaged history that emerged in our project was history as celebration. This notion was buoyed by the events surrounding the 70th Anniversary of the NHS in July 2018, which the historian Andrew Seaton positioned within a longer term shift in cultural narratives of the NHS ‘from caution to celebration’.^15^ In part, this anniversary gave our project renewed momentum and exposure. Roberta Bivins was able to build a partnership with the production company 7Wonder, through which our team advised on a three-part BBC Four documentary, *The NHS: A People’s History*.^16^ While our team provided historical context, information and suggested interviewees, the producers provided further content for our website. The producers also asked all of their interviewees our key research questions: what is your first memory of the NHS? Where will the NHS be in 70 years? What would you do for the NHS? What does the NHS mean to you?^17^ The producers found brilliant interviewees, for example, the Bellringer Babes, women who had had gender reassignment surgery, and who created badges to honour the seven NHS surgeons qualified to perform this work. Dawn and Jenny-Anne discussed the lifelong emotional attachments that they had formed to their surgeons, who, they stated, saved their lives. They manifested this emotional attachment through the material culture of their pin-badges, as well as through their friendship and media interview.^18^ For Dawn and Jenny-Anne, these interviews were an opportunity to celebrate their NHS surgeons and to highlight this significant issue. Such interviews—and those with other patients, campaigners and staff—added new and diverse perspectives to our project research and to broader cultural narratives of the NHS, amplified by the influential channels of the BBC.^19^

During the Anniversary, we were also able to work alongside the commemorative plans of NHS England, which sought to co-ordinate national and local efforts. This meant that we gained awareness of other NHS history projects, organised by academics, local historians and staff and patient groups, as well as further media exposure. In this mass moment of interest in the NHS, project members were able to discuss their specific research interests with relevant audiences. Natalie Jones, for example, a Public Engagement Research Fellow, presented her research on Aneurin Bevan, the Minister of Health when the NHS was founded, to the Welsh People’s History Society. Jane Hand, a Research Fellow, discussed her research into the visual culture of public health films at the British Film Institute, as they launched a new online streaming service, ‘NHS on Film’.^20^ I was invited to discuss work on NHS hospital food—previously conducted with Margaret Charleroy, researching institutional diet in prisons—on the Channel Four programme *Sunday Brunch*. Indicative of the broader stimulation from such engagement, three historic hospital dishes were cooked for this appearance: an egg and tomato jelly, from a December 1948 King’s Fund recipe book; a fish custard from the 1950s, produced by the Royal Infirmary of Edinburgh and curated by the Lothian Health Services Archive; and a red wine burger, published in a renal diet manual by the King’s Fund in 1982.^21^ While my previous research had looked at the cultural representations—and indeed stigma—around hospital food, this collaboration provided visual and textural clues as to how such food was produced in history—areas increasingly of interest within emergent work on the sensory environments of health care by, for example, Victoria Bates and Claire Hickman.^22^ The assertion by musician Plan B that the tomato and egg jelly tasted like Heinz’s tomato soup and was ‘all right, I’m into it’ demonstrated the ways in which actually constructing and *tasting* this food could challenge our contemporary assumptions, born from its textual descriptors.^23^

In general, therefore, the celebratory narratives around the NHS anniversary gave our project further opportunities. Primarily, these were opportunities for *dissemination* rather than for interactive engagement. These were opportunities that, like Anniversary culture more broadly, may themselves be celebrated in an age focused on academic ‘impact’ and ‘value for money’.^24^ Nonetheless, there was also the ongoing risk that participating in anniversary celebrations would involve us reiterating and bolstering simplistic cultural narratives and reframing our research in these terms. Historians of culture have been critical about the lack of ‘dynamism’ researchers have brought to anniversary work and have further questioned, indeed, whether academic scholarship, for example, around the First World War, may be ‘powerless’ in the face of cultural interpretations, such as around popular television programme *Blackadder*.^25^ Certainly, our project encountered the strength of existing historical narratives around the NHS, which many journalists wanted to reinforce and reproduce. Media inquiries during the Anniversary requested advice about historical ‘milestones’ or timelines, suggestive of a narration of history as ‘70 achievements for 70 years’ or as defined by the ‘greatest’ or ‘best’ or ‘most tragic’ events. Within these suggested narratives, the past was flattened and sensationalised, and it was difficult to maintain a vision of the past as ‘complex, often messy and contingent’.^26^ Apparent ‘celebrations’ were also to an extent condemnatory: looking to restrict historical debate or to reiterate a specific—and preconceived—series of narratives. These narratives were bound by popular periodisation that historians look to complicate.^27^

Aware of these risks, our project sought to offer critical narratives of the celebratory anniversary moment, looking to echo the argument of historian Tim Grady that historians may be able to add more diversity, new narratives and critical thinking into anniversary moments.^28^ To give one example: to mark this anniversary, the *British Medical Journal* ran a poll asking readers to comment on the NHS’s ‘greatest achievement’. The survey received 5,550 votes, 23 per cent of which voted for its ability to serve those with the ‘greatest need’. Looking to add complexity to this vision, I pitched to, and subsequently wrote two blog posts for, the *British Medical Journal Opinion* website. Looking to problematise this framing, the first article pointed to how interest in tracing the NHS’s ‘achievements’ had increased over time, citing work from Roberta Bivins and Nick Hayes about the lack of public interest in the NHS at its inception, with press ‘silent’ and ‘no great popular demand from the average man or woman in the street for radical or overarching change’.^29^ The second article, responding to the survey results, discussed a history of activist and public critique around defining ‘greatest need’, with second-wave feminist and health campaigners, for example, having argued since the 1970s and 1980s that the ‘needs’ of women and minority communities were forgotten first during cuts and closures.^30^ These contributions were, of course, relatively small, but nonetheless provided an indication of how we sought to respond to media interest in celebratory cultures while also using history to complicate and nuance views of current issues. In this framing, our historical research itself became a source, which looked to make historical activist critique visible and potentially powerful.

Indeed, as we continued this work, we increasingly found that many partners were keenly receptive to such critical interventions. In this anniversary moment, contrary to our expectations, a small but significant strand of policy and media partners was raising parallel questions about ‘Anniversary fever’. The *Financial Times* in the Netherlands, for example, contacted our project while researching public love for a Service inundated with ‘beeps and creaks’, raising interesting questions about the distinct national cultures of socialised medicine.^31^ The papers’ final article cited our project’s research on how this public love—or the vision of the NHS as ‘religion’—had become particularly powerful from the 1980s, entwined with a developing sense of potential loss under the Thatcher governments. At public events, also, we found evidence for the historian Dan Healey’s assertion that ‘lay audiences have an appetite for the serious, the engaged and the challenging in historical argument’.^32^ Grace Huxford and Richard Wallace, likewise, reflecting on their oral history project around the 50th Anniversary of the University of Warwick, found that interviewees sought to ‘tell an alternative institutional history’ and to reflect on who owned and retold institutional memories.^33^

One common memory shared with our project discussed the trauma of extended hospital stays, particularly in childhood, and the long-term impact of these memories. We were contacted, regularly, in person and online, by participants who shared negative memories and suggested that they did not expect us to use, consider or share these. This demonstrated that while we may assume that publics will *only* want celebratory narratives of history, in fact, members of the public, in turn, may assume the same of historians. By hearing memories with an open mind, we learnt that participants shared our goal to complicate and nuance NHS history, and that we could provide them with spaces or encounters through which to discuss and contextualise their thoughts. Oral historians have shown the potential for such interpersonal discussions to inspire reflection, understanding and even acceptance for those involved, as well as to ‘give voice’ to groups whose stories have not always been heard.^34^

## Collaboration

When working collaboratively, our project had the most benefits for involved historians and publics. Nonetheless, this type of collaboration was challenging and time-consuming. This can be demonstrated through examination of how the NHS project informed my research into post-war childhoods and welfare. This topic became a key theme on our website and thinking, shaped by and entwined with public responses. Indicating this, two of the most popular blog posts on our website were on ‘Childhood Vaccination and the NHS’ and ‘Premature Birth and the NHS’, both written by Jane Hand.^35^ Childhood also emerged as a key theme at public events, with people keen to recall their childhood glasses, memories of vaccination or lost items. At Leamington History Society, for example, one participant discussed a purse made, while a child patient and another described a diary they were forced to keep during a hospital stay. Both of these fascinating items, revealing of a history of enforced creative production within institutions, had been lost, like many objects we heard about, suggestive of a hidden and irretrievable material culture of patienthood.

My previous published work on childhood and health sought to explore how constructs of children’s experiences had become increasingly powerful in public policy and media discussions over the late twentieth century, and particularly from the 1970s and 1980s, entwined with public participation and social movements and growing focus on children’s psychological and medical consent.^36^ This research maintained that, while we can trace the politics of children’s agency and experiential expertise, we could not—as Carolyn Steedman argues—‘speak for’ nor trace the content of these experiences.^37^ Nonetheless, the NHS project provided a space through which we could begin to think through children’s agency and experiences in two key ways: by looking at adult memories of childhood experiences and by looking at the art work of contemporary children and young people. Different notions of experience were traced through diverse sets of sources: memories from adults, young people and children, produced online and in person, and visually and textually. Examining these demonstrates the variety of sources that public engagement may generate—material culture, hidden papers and documents and lengthy and fleeting oral and written testimony.

The first revealing source was adult memories. Without us explicitly soliciting this, one of the most common memories and comments shared on our website surrounded adult memories of childhood long-term hospital stays, notably in the tuberculosis wards common in the 1950s. The memories in this area showed how typical childhood experiences were inflected and rewritten by experiences of disease and institutionalisation. Writing about this, one contributor to our website not only recalled the joys of Christmas on the wards but also being ‘plonked’ onto Santa’s lap rather than able to walk there. Others recalled watching children’s television in hospital, enjoying the smell of clean laundry and receiving favoured childhood snacks—chocolate buttons and cookies with icing. Yet entwined with these memories, authors also recalled the loneliness and isolation of hospital wards, not being allowed to play with toys and the lack of parental contact, as parents were not typically permitted to visit. Our open digital space, simply inviting anyone to contribute their ‘NHS story’, enabled people to share their own individual narratives, memories and beliefs in a range of formats and styles.

The second revealing source was the drawings by children and young people, produced at engagement events, for example, our Roadshows, with child hospital patients, and at an outreach course. In the former two settings, we provided art materials and asked children to ‘Draw Their NHS’, hoping to use these materials to unpick how, exactly, children viewed the Service, and indeed whether they recognised a distinctive ‘NHS’ or merely the work of medicine more generally. In the materials that resulted, children most commonly drew medical transport: helicopters and ambulances. In St Fagans, one child drew a stick person that, an adult later wrote, represented the fact that, ‘the NHS is a superhero’. Other key themes were pictures to represent bacteria and disease. Children also drew stick drawings of specific medical staff, often male doctors and female nurses, echoing broader historical representations of gendered roles within the health service.^38^

In another engagement activity, designed by Mathew Thomson, we asked secondary school students attending an outreach course at the University of Warwick to draw what a Museum of the NHS would look like. These pictures demonstrated that the students had relatively strong knowledge of NHS history: with Aneurin Bevan a regular feature. They also began to break down key chronologies of the NHS, organising, for example, by theme—from birth to death (cradle to grave) or from decades periodised from 1948 to 1958, etc. Multiple drawings revealed concern about the present and future of the NHS, featuring spaces discussing this, and planning to acquire the bus, made famous during the campaign around the EU referendum of 2016, which suggested giving £350 million per week to the Service.

We therefore generated very different types of source—all relating to ‘youth experience’ but reflecting on these experiences through reference to personal memories, stories, comments and drawings and at different locations and phases of the life course. Nonetheless, looking at this diverse range of sources—as was only possible through public engagement work—began to give us access to a new history of meanings and feelings. Analysing children’s drawings produced during the Holocaust, Nicholas Stargardt has argued that these reveal ‘clues as to how children’s artistic imaginations were formed’, as children would use their drawings to try and understand how systems worked within concentration camps and as a source to channel their emotions.^39^ More broadly, all of the sources produced through our engaged research—verbal and written accounts of memory, brief or lengthy conversations and drawings—helped us to think about how young people had formed relationships with the NHS over time. These sources represent a significant resource—rarely found in the archives—for the emergent and developing child-centred histories being written by, for example, Sian Pooley, Laura Tisdall, Catherine Sloan, Hannah Elizabeth, and Sarah Kenny.^40^ Furthermore, these sources push historians to take young people’s testimonies as significant on their own terms and to value thinking about how children engage with welfare systems *as* children. Pooley has argued that this approach is rare in existing historiography, which, in part driven by the types of sources primarily available, tends to instead consider young people’s actions solely as indicators of who they will become.^41^

Most obviously, this engaged research suggested that young people did and have recognised the NHS as a distinct object of analysis, as opposed to merely noticing ‘medicine’ or medical treatment. Students at the summer school, indeed, centred one museum around a fountain of holy-water, recognising the connotations of the NHS as religion, or the ferocity of public attachment to this institution or name. Even the youngest children participating in our drawing activities recognised the activity when asked to, ‘draw the NHS’, showing the power and prevalence of this acronym. Furthermore, in terms of thinking about the meaning of this institution, its mundanity and everydayness were also clear from the children’s drawings, which put such objects as glasses, uniforms and teeth as central. This suggests young people’s interest in taking analysis of health care and welfare beyond the clinical encounter or even beyond hospitals—often a key focus of social histories of medicine—towards households, leisure spaces, schools and communities. Roberta Bivins, Hilary Marland and Nancy Tomes argued in 2016 that examination of the household, in particular, enabled historians ‘to explore vital aspects of preventive and therapeutic activities that are often overlooked in grand narratives of scientific, market and professional change’.^42^

While tracing medical intervention as commonplace, and ordinary, adult memories also showed the distinct ways in which universal health care for children had reshaped lives for subsequent decades, for example, by recalling different treatments before and after the inception of the Service or reiterating an ongoing fear of losing access to the NHS. These diverse sources, then, began to show us how early that young people are—and were—socialised to ‘learn’ and understand the NHS.^43^ These sources also suggested how childhood perceptions of health care systems emerged: through parental contact—note the addition of the ‘NHS as superhero’ narrative atop a child’s drawings and through contact with medical professionals, peers and current affairs, all charted in multiple memories and conversations. Recognising the range of influences over everyone’s views of ‘the NHS’, our activities were designed to enable young people to explicitly reflect on and share their own perceptions of the Service. While many welcomed this invitation, others subverted or rejected the designed activities, for example, by refusing to participate or by drawing scribbles, doodles or alternative images that did not seek to represent ‘the NHS’. As engaged researchers, this invited us to think again about the activities we had designed and to be self-critical about how our events could be more interesting, varied and inclusive.

More broadly, such engaged research encouraged us to consider how the categories of age and generation subtly nuance any simplistic cultural or political vision that ‘everyone’ loves the NHS. In terms of age, and echoing findings by recent social surveys, we found that generation was a significant factor in shaping public feelings about the NHS, with older populations, who recalled family memories about the foundation of the Service in 1948, most likely to defend the NHS specifically. Younger generations, meanwhile, were more likely to raise concerns about a perceived decline across a broader post-war social settlement composed of education, social services *and* health, within which the NHS was only one component—albeit a highly symbolic one.^44^ This finding chimes with research in war studies around how cultural perceptions shift as those with lived memories pass; ‘signal figures’ from wartime generations, like those born around the inception of the NHS, offer a perception of ‘authentic experience’ that reshapes public debate and may function to inhibit or encourage ‘critical evaluation’.^45^ Such research demonstrated the significance of working with people of all ages during research projects, and of giving voice, in particular, to the perspectives of the very young and the very old.

Thus childhood memories or experiences, organically generated following the work of our project, gave rich textual and emotional details to existing histories of policy change around childhoods in hospital, adding to existing sources from archives and oral history. In this context, archival sources are very valuable, but their focus on institutional records, clinical data and patient photographs can be greatly enriched with the addition of personal testimony.^46^ Engaged research enabled us to think closely about how *all* childhoods were richly and emotionally textured by illness, disease and health—this enriches historiographies of childhood disease, which may focus because of availability of clinical sources, on examination of children whose lives were dominated by sickness and disease, rather than on those who encountered illness on a fleeting, daily level and whose experiences were thus not typically documented.^47^ Such engagement also moved beyond many archival sources in providing evidence of the long-term nature of childhood memories and the resonating and emotional impact of childhood experiences. As Hannah Newton has argued, historians can find significant access to children’s voices in the context of disease as ‘parents and relatives recorded the thoughts, words, and actions’ of their sick offspring ‘in detail’ in early modern England, at least, ‘driven by awareness that these might soon be cherished as last memories’.^48^

This type of work raised new questions for our research, which had not been generated by our archival work, and enabled publics to create new archival sources for ourselves and for future historians. The memories mentioned earlier, for example, may be interpreted in different ways in future years. More broadly, during our public events and on our website, we received photographs and stories that produced new textual and material accounts of activism, illness and well-being. For my own work, this is already having an influence. Dialogue with Leeds Hospital Alert, a campaign group established in 1981, and London Health Emergency, established in 1983, led to new resources being deposited on our project website and in the Modern Records Centre, Coventry, and also challenged my interpretation of how region shaped activist cultures. As historians and archivists have powerfully argued, the construction of archives—what is kept and what is lost—reflects existing dynamics of power and structure, shaped by available funding, space and resources.^49^ In this context, everyday collaborations with a diverse range of publics can, like oral histories, enhance the future conditions under which knowledge will be produced, enabling publics to add new documents, as well as new questions, to archival and published records.^50^ Trying to write about experience, meaning or feelings, but without making this kind of human contact, seems to be missing vast opportunities for analysis, as well as potentially in part suggesting a false dichotomy between two types of scholarly method: the examination of archival sources, being constructed as ‘valid’ and ‘objective’, and public engagement, perceived as subjective, unreliable and too inflected by bias and chance for scholarly use. As oral historians have aptly demonstrated with regards to interviews, no such divide is clear.^51^

## Challenges

Thus the Cultural History of the NHS project sought to forge a model of critical collaboration, rather than celebration or condemnation, in engaged research. It sought to nuance debate around the NHS anniversary while also looking to gather information from public engagement and archival material as sources for our own and future research. This work was not without challenge. Reflecting on these challenges is key to foregrounding future work in this area, to protecting the emotional health of those who conduct engaged work and to recognising the ‘inevitable constraints’, as well as benefits of participatory approaches.^52^ The first challenge in this work—which seems mundane but was significant—was lack of interest. Echoing the kind of lack of interest that Bivins and Hayes have uncovered around the early NHS, indeed, we encountered many people—at events, through our website, by email—who were uninterested in our project and in thinking about NHS history.^53^ In part, this was an important reality check, reminding us that, of course, not everyone would be interested in NHS history, and that we must work hard to design activities that are engaging and beneficial for participants. At the same time, meeting apathy while trialling such activities experimentally is difficult and tiring. It is tiring to stand by a stall for a day, asking, ‘would you like to contribute to a history of the NHS?’, where about a third of those walking by state, with great confidence, ‘No!’, or simply laugh.

Likewise, many of our experiments failed or struggled, which was challenging. Notably, in our online community, members of the public engaged with us, and with website content, but did not talk to one another in the ways that we had initially hoped.^54^ Indeed, and demonstrating the contact behind such a website, looking at the first 69 memory submissions, I uploaded over a third, after substantial contact via email with members of the public who were reluctant or unable to upload these themselves. Surveys that we received at public events, likewise, were sometimes the result of furious writing, but at other times reflected conversations between visitors and our team, or, perhaps, even coaxing and suggestion. Engagement work was derailed not only by lack of interest but also by external factors: multiple conversations about the NHS turned to Brexit; an event at Rugby Art Gallery faced very poor turn out as it coincided with strong wind storms. These kinds of encounters are not only part of the experimental learning of engaged research but are also personally confronting and physically very tiring.

When members of the public did excitedly engage, and when we began to meet the ideals of collaborative history, we met the challenge of how—and whether—to position ourselves, our own memories and beliefs, within this interaction. Talking with members of the public in open ways about our own lives, views and relationships with the NHS would often improve our conversations. Bringing my own NHS glasses, for example, which I wore as a child, encouraged members of the public to tell me their own childhood stories. Like those who brought objects to our Roadshows, my parents had carefully curated my first pair of glasses, for my future reference, and could retrieve them on my behalf when I started work on this project—positioned within the kind of ‘family archive’ examined by Anna Woodham, Laura King, Liz Gloyn, Vicky Crewe and Fiona Blair.^55^

Concerns from academic conferences, where we discussed such work, were often around whether such engagement work may push our scholarship towards the present, meaning that we had present-centred discussions at events and obscured views of the past. Nonetheless, however, and as oral historians have amply demonstrated, discussions with members of the public frequently discussed change over time. Childhood glasses evoked memories of youth and its relationships with material cultures, care and health. Stigma was also a key theme in discussions of childhood glasses, and the idea that the distinctive NHS frames provided a visual label and identity for poorer children.^56^ The most frequent narrative emergent from our public events, without prompting, was that nurses’ uniforms had shifted from formal to casual, reflecting a perceived broader lack of deference and hierarchy in the Service over time. Campaigners, meanwhile, would also often discuss historical change, arguing that successive reforms had brought privatisation to the NHS, particularly from the 1980s. Participants were often interested in how these individual memories could be contextualised and situated within, but also enhance and extend, broader histories. For example, public memories about children’s stays in tuberculosis wards, which we received, could be located in a specific mid-century historical moment. These memories immediately followed the introduction of the NHS, from 1948, but were less likely a product of the late 1950s onwards, as vaccinations for tuberculosis were introduced for children of 14 years in 1953.^57^ The mentions of restricted parental visiting, meanwhile, were likely situated in a 1940s and 1950s context: Alex Mold has shown that restrictions became more lax from this time, following parental campaigning and growing psychological interest in attachment.^58^

Thus, providing historical context meant that we were able to trace a range of public feelings and beliefs around a relatively time-bound phenomenon. Situating these public memories could map onto, and at times critique, historiographical narratives: of shifts in NHS policy or changing cultures of privacy and secrecy.^59^ Indeed, as Miri Rubin has argued with regards to presentism, reflection on the present, or ‘controlled anachronism’, may help us to reflect on our own views, to accept the contingency of the past and to read against the grain.^60^ Alexandra Walsham, furthermore, has questioned whether a critique of presentism is a relic of a vision of history as ‘objective’, inherited by the positivists of the nineteenth-century discipline and denying the legacies of second-wave feminist and post-colonial histories and campaigning.^61^ Indeed, it was my view that we entered public events not only as ‘researchers’, looking to study those attending, but also as members of the public, patients of the NHS and participants in local communities. As Ann Oakley has demonstrated in relation to oral histories of new mothers, people will respond to interviewers with reference to all of these identities—whether we invite it or not. By embracing this kind of personal interaction, we strengthen meaningful engagement, avoid the imposition of hierarchies between ‘researcher’ and ‘researched’ and access qualitatively rich detail.^62^

Overall, entering engagement events with openness was beneficial for the people we engaged with and the histories we wrote. Nonetheless, this type of engagement, and the blurring of personal and professional lives, did require a specific and intense kind of emotional labour. The emotional labours of all historical work—applying for grants, responding to peer review, developing field work, interpreting data and disseminating findings—have been recently made visible in a powerful new edited collection by Tracey Loughran and Dawn Mannay.^63^ As this collection makes clear, these emotional relationships shape the research that we produce and the lives that historians lead, and yet descriptions of these processes have rarely had a ‘visible presence on our bookshelves and in our libraries’.^64^ For involved publics, likewise, Wendy Rickard’s work emphasises that oral history interviews can be ‘draining’ for participants, particularly for those discussing ‘traumatic or taboo experiences’, as well as potentially ‘affirming’.^65^ Engaged research around social histories of medicine—this highly personal and emotive topic, which touches on everyone’s lives—is likewise prone to evoking individual reflection and emotion for all participants.

Indeed, in researching this area, workers on our project were not always able to clearly blend our personal and professional lives: these things could also conflict. At times, members of the public at our events made comments about the NHS, which we politically disagreed with, for instance, in terms of its relationship with immigration. As a young, female early career researcher, I sometimes faced the challenge of having to respond to this, looking to counteract these views, with people who were both relatively unengaged with my project but also holding views that I found deeply troubling. The emotional burdens of engaged research in the social history of medicine may, as in other disciplines, fall particularly along gendered lines, as Mary Morris and Andrea Davies have emphasised.^66^ Media studies theorist Heather Savigny, indeed, has argued that the impact agenda functions to expose women to ‘structural and symbolic violence’, particularly online.^67^ The production of public engagement activity, therefore, places emotional burdens of organisation and co-operation onto researchers, which in archival work are in part shared with, or primarily borne by, archivists and curators.

Taking another case study, my research on this project around campaigning in defence of the NHS further showed the potential for our engaged analysis to effect the emotions and politics of ourselves and our participants. In exploring health activism through oral history, survey materials and archival research, my work uncovered multiple forms of solidarity forged between health campaigners. My research also made visible areas of tension and disagreement faced by and within campaign movements. In a survey of 175 campaigners, self-identified online, respondents disagreed, for example, about whether the dynamism of this movement benefited from close social ties and networks, or whether this may make it appear too exclusive, with the same people caring about the same issues.^68^ Archival materials revealed further contested questions—for instance, materials produced by 1970s and 1980s campaigners revealed ambiguities around how to defend ‘the NHS’ and universal health care while also critiquing perceived declining standards in specific hospitals, which they traced to insufficient funding.^69^

Important engaged research into second-wave feminism has likewise uncovered and explored tensions within this movement. Lucy Delap, for example, using archival and oral history research, has analysed the contested position of anti-sexist men within the feminist movement and the ways in which these men sought to reconcile ‘their leftist critique of the nuclear family … with the intense feelings they had for their children’.^70^ Analysing a participatory workshop in 2013, bringing together feminist activists and academics, Sarah Crook and Signy Gutnick Allen argued that there were ‘tensions exposed in the sessions’, notably across generations of feminists.^71^

While historians are typically interested in probing such complexities and contested interactions, the significant research of Delap, Crook and Gutnick Allen raises several points for reflection, which prove invaluable in analysing the NHS project. Delap argues that anti-sexist men ‘experienced a powerful affect – that of shame – that they were not able or willing to name’.^72^ From this study, she asks: ‘How appropriate is it to read unconscious affects into the anecdotes and embodied experiences of historical actors?’^73^ This raises the questions of how historians should interpret and represent the experiences we elicit at public engagement events. More broadly, this analysis also provokes reflection on how engaged research may generate and reshape participants’ thinking. Further exploring this idea, Crook and Gutnick Allen argue that the boundaries between academia and activism are ‘porous’ and provide an example of how activists’ practice in documenting their work has been shaped by imagined interactions with future historians and the understanding that ‘they themselves were making history’.^74^ While there are no singular frameworks to govern the relationships between academia and activism, this research—and the experience of the NHS project—demonstrates that we must not only ask how research may *represent* activisms. Engaged researchers must also grapple with the complex questions of how our work and our partnerships may *change* the emotional states and political engagement of our participants and ourselves.

Thus engaged research forces us to confront our political, professional and personal aims and to reflect broadly on the potential effects of our historical research. As Chris Millard has argued, we must also think critically about the valorisation of ‘experience’, building on the disciplinary assumptions of psychoanalysis, social history and anthropology, all of which have left invocations of ‘experience’ as seemingly irrefutable.^75^ While historians of medicine, and particularly of more distant periods, may feel able to interrogate and challenge archival sources, we are nervous to do the same when confronted directly with the authors of public testimonies, who may resent our interpretations, withdraw their consent to participate or even indeed reject the characterisation of their words as a ‘source’.

This important intervention by Millard raises questions of how we can best harness insights from the tensions and collaborations wrought by engagement. In part, perhaps, reflecting on challenging collaborations pushes us to think about how historians and participants in research alike are guided by our ‘experiences’ and our ‘expertise’. Discussing conflicts and tensions we face as engaged researchers—in personal peer groups and academic journals—makes the working conditions of our analysis visible, drawing on traditions of feminist and oral history research. In particular, such work aides analysis of how a researchers’ own position inflects their work and its reception, actively confronting the idea that ‘what can be known can only be known through oneself, one’s lived experiences, and one’s biography’.^76^ It is potentially also powerful to confront these tensions themselves in partnership with activist, community and public groups and to recognise that this is not only a conflict uniquely faced by historians. Rather, the dissonance between professional, personal, political, familial and community agendas is one faced in varying ways by everyone, and this was certainly also something that came to the fore in our NHS history community workshops, where community mediators and attendees often spoke as people who had experienced health care, community figureheads, mediators, friends and family members. Potentially, destabilising the idea of historical practice as ‘special’ or ‘unique’ is significant here.

## Conclusion

The Cultural History of the NHS project showed the potential to utilise and analyse public engagement as a method: both in terms of historians *providing* sources that look to reshape cultural understandings and in terms of historians *generating, storing* and *keeping accessible* new sources from events, websites and ‘family archives’.^77^ The project aimed to echo models of critical and collaborative research, which genuinely involve the public in forming research questions and reframing focus, and avoid narrow visions of history as condemnation or celebration.

This model was based on the following three related premises. First, it rejected the classification of historians as providers and publics as consumers of knowledge and rejected the vision of a ‘public-expert gap’.^78^ Rather, following oral histories, feminist histories, co-produced histories and histories ‘from below’, we recognised a model whereby everyone has ‘knowledge’, of various types, and assumed that, while some have more relevant skill sets, interests and labours invested in writing history, the conditions under which history is produced, archived, curated, stored and remembered extend far beyond academic historians alone. These historiographical traditions also offer significant precedent for recognising testimony, drawings and fragments from engagement events as valuable sources, despite the recent nature of their generation.

Secondly, and building on this point, this model recognised that there are different types of public participation in social and policy life, building on a model established by Ellen Stewart in terms of how to understand public participation in health reform.^79^ In this context, therefore, we must understand official and unofficial, wanted and unwanted, illicit and licit public participation to understand change over time. Likewise, when planning our public engagement, we must recognise that we engage with a dynamic set of publics who will look to accept, challenge, renegotiate and reshape our research through a variety of mechanisms. In doing so, we must also reflect on the ways in which our collaborative work can facilitate or obscure public action, not merely look to interrogate an imagined ‘the public’ as a source-base, and move on. Thirdly, and finally, this model called for a shift, echoing Alix Green, from an academic and evaluative emphasis on historical ‘content’, what we produce, our ‘outputs’, to process, looking at how we work. While Green has argued that this shift is key to thinking about how historians work with policy, this also shapes how we can work with publics and each other.^80^ A broader shift towards thinking about how history is constructed is evident in Green’s new project about the construction and utility of business archives and also in the Working Knowledge project at the University of Durham.^81^

Constructing collaborative and engaged research, and analysing engagement as a method, has significant challenges, which we must reflect on honestly, drawing on the reflexive practices of feminist historians and sociologists.^82^ These challenges include: emotional fatigue—the significance of which should not be underestimated; the negotiation of our political positions when confronting uncomfortable memories or viewpoints; and lack of interest. These challenges are magnified within academic systems reliant on fixed-term labour, meaning that institutional memory around engagement, and peer support for engaged researchers, can potentially be lost to departments. This raises a key question, however, in terms of whether historians are expecting archivists, at present, to burden the challenges of acquiring and processing sources alone, while historians themselves then benefit from accessing these. Likewise, while we increasingly have public engagement professionals working at Universities—and often as key figures within large-scale medical humanities grants—it is imperative that historians do not merely discharge the emotional labour of this work to managerial and professional members of their teams, or indeed to junior female scholars, and thus replicate the kinds of hierarchies that engaged research should look to destabilise.

Thus utilising public engagement as a method encourages social historians of medicine to rethink and enrich multiple historiographies—this article began to establish links, which will be pursued in later research articles from this project, between our engagement findings and histories of childhood disease, generational change and activist feelings towards the NHS. Yet, *reflecting* on the purpose and vision of engagement as a method, in the same way as we do with oral histories and archival sources, also encourages social historians of medicine to reflect on how our own experiences of society and health shape what we write, how we collaborate and the models of knowledge that we prioritise.

Within this model, historians must reflect on our own limitations, entwined with the limits of our sources, and also on the potential limitations of our use and expertise in certain community and engagement settings. While requiring great humility, we must recognise, as Des Fitzgerald and Felicity Callard have cautioned, when to leave engagement projects because we are not the most relevant or appropriate actors to progress them.^83^ Engagement is not a magical and limitless source-base, which can be used to further any research project or agenda or to answer any questions that archives leave unsolved. Nonetheless, the Cultural History of the NHS project was constantly surprised at the range, depth and intimacy of topics that participants in our events were keen to discuss. Participants in our project actively negotiated how to ensure that working with us, or sharing their experiences in a fleeting manner, was empowering, interesting or useful for them or provoked an active challenge to our research and analysis of our own roles and responsibilities.

Overall, the Cultural History of the NHS project has further demonstrated the value of writing critical and collaborative histories and of utilising engagement as a method and resource for research and analysis. While tempting to conclude that this is only relevant for modern social histories of medicine, where living audiences were easy to encounter, this article contends that this is the case for all historical scholarship. Notably, the processes of reflecting on our knowledge production, our status as expert, the questions we raise, the ways in which we process and acquire data enhance all research. Thinking about what kind of universities we want to work in and the ways in which researchers are conceptualised as detached expert, or as providers of knowledge, again, affects everyone’s working practices.^84^

## Footnotes

1 Powerful social historical accounts that utilise personal experience include: Carolyn Steedman’s *Landscape for a Good Woman: A Story of Two Women* (New Brunswick: Rutgers University Press, 1987); Alison Light, *Common People: The History of a Common Family* (London: Penguin, 2015); Jon Lawrence, *Me, Me, Me: The Search for Community in Post-War England* (Oxford: Oxford University Press, 2019). In terms of radical histories, see, for example: Raphael Samuel, *Theatres of Memory: Past and Present in Contemporary Culture* (London: Verso, 1994), 8; Onni Gust, ‘What is Radical History Now?’, *History Workshop Journal*, 2017, 83, 230–40. A defence of oral history as combining methods with the products of research is laid out persuasively and clearly in: Lynn Abrams, *Oral History Theory* (London: Routledge, 2010). Further bodies of literature assessing the production and significance of oral history are very large and important, and useful starting points are the journals *Oral History* <https://www.ohs.org.uk/journal/> (accessed 13 August 2019) and *The Oral History Review* <https://academic.oup.com/ohr> (accessed 13 August 2019). An example of one institute that studies and analyses co-produced research is the Brigstow Institute at the University of Bristol, see descriptions of their recent projects at: ‘Projects’, *Brigstow Institute*, <http://www.bristol.ac.uk/brigstow/projects/> (accessed 4 September 2019).

2 See on these trends: Harry M. Collins and Robert Evans, ‘The Third Wave of Science Studies: Studies of Expertise and Experience’, *Social Studies of Science*, 2002, 32, 235–96 ; William Viney, Felicity Callard and Angela Woods, ‘Critical Medical Humanities: Embracing Entanglement, Taking Risks’, *Medical Humanities*, 2015, 41, 2–7; Rhodri Hayward, ‘Review: “Much Exaggerated”: The End of the History of Medicine’, *Journal of Contemporary History*, 2005, 40, 161–78; Havi Carel, ‘Breathlessness: The Rift Between Objective Measurement and Subjective Experience’, *Lancet Respiratory Medicine*, 2018, 6, 332–33; Havi Carel, ‘Invisible Suffering: The Experience of Breathlessness’, in Lenart Škof and Petri Berndtson, eds, *Atmospheres of Breathing: The Respiratory Questions of Philosophy* (New York: SUNY Press, 2018), 233–46; Havi Carel and Tina Williams, ‘Breathlessness: From Bodily Symptom to Existential Experience’, in Kevin Aho, eds, *Existential Medicine: Essays on Health and Illness* (London: Rowman & Littlefield, 2018), 145–60; Ann Oakley, *Fracture: Adventures of a Broken Body* (Bristol: Policy Press, 2007).

3 The Wellcome Trust and UKRI—key funders in this field in the UK context—both encourage that public engagement is included within funded projects, from inception to evaluation, and at all career stages. To read about the Wellcome’s approach, please see: ‘How we engage the public’, Wellcome <https://wellcome.ac.uk/what-we-do/our-work/public-engagement> (accessed 12 August 2019). To read about the UKRI approach, please see: ‘Public engagement’, UKRI <https://www.ukri.org/public-engagement/> (accessed 12 August 2019).

4 See, for example, the engaged research work of recent Wellcome Trust-funded projects on *Surgery & Emotion* <http://www.surgeryandemotion.com/> (accessed 4 September 2019); *Placing the Public in Public Health* <http://placingthepublic.lshtm.ac.uk/> (accessed 4 September 2019); *Body, Self, and Family* <https://bodyselffamily.org/> (accessed 4 September 2019); *Sex and History* <http://sexandhistory.exeter.ac.uk/> (accessed 21 November 2019).

5 A discussion of the National Institute for Health Research approach to public and patient involvement is set out here: ‘Guide for Researchers working with Patient and Public Involvement (PPI) Contributors’, <https://www.clahrc-oxford.nihr.ac.uk/files/get-involved/guide-for-researchers-in-working-with-ppi-contributors-17.pdf> (accessed 12 August 2019). The *British Medical Journal*’s description of how to report patient and public involvement in research is here: ‘Reporting Patient and Public Involvement in Research’, *Medical Humanities* <https://mh.bmj.com/pages/authors/#reporting_patient_and_public_involvement_in_research> (accessed 12 August 2019).

6 One significant new resource for analysis of public engagement as a method is the new journal *Research for All*, which was established in 2017 and has had three issues thus far, providing significant case studies from engaged researchers and engagement professionals: *Research for All*, <https://www.ucl-ioe-press.com/research-for-all/> (accessed 13 August 2019). The Working Knowledge series in related ways looks to make contexts of research production visible: *Working Knowledge*, <http://www.workingknowledgeps.com/?page_id=223> (accessed 13 August 2019). A useful model in terms of analysing public engagement for a social history of medicine project is: Janet Weston, ‘Oral Histories, Public Engagement and the Making of Positive in Prison’, *History Workshop Journal*, 2019, 87, 211–33. Another important article in this area is: Laura King and Gary Rivett, ‘Engaging People in Making History: Impact, Public Engagement and the World Beyond the Campus’, *History Workshop Journal*, 2015, 80, 218–33.

7 Martin Gorsky’s review of 2008 observes the lack of cultural histories of the NHS: Martin Gorsky, ‘The British National Health Service 1948–2008: A Review of the Historiography’, *Social History of Medicine*, 2008, 21, 437–60. Further quotes from the project’s institutional website: ‘Cultural History of the NHS’, University of Warwick, <https://warwick.ac.uk/fac/arts/history/chm/research/current/nhshistory/> (5 March 2019).

8 These research fellows were in alphabetical order by surname: Dr Jennifer Crane, Dr George Gosling, Dr Jane Hand, Dr Natalie Jones and Dr Jack Saunders.

10 Martin Wright, ‘People’s History’, in Tracey Loughran, ed., *A Practical Guide to Studying History: Skills and Approaches* (London: Bloomsbury, 2017), 81–85.

11 Discussing the role of digital source-bases in shaping knowledge, see: Julia Laite, ‘The Emmet’s Inch: Small History in a Digital Age’, *Journal of Social History*, Advance access online 11 February 2019, https://doi.org/10.1093/jsh/shy118.

12 While this article does not have space to address this aspect of the project in detail, material culture is a rich and vibrant new area of scholarship. Examples of this type of work are present in the *Journal of British Studies*’ ‘One British Thing’ series, for example: Beverly Lemire, ‘One British Thing: Clay Pipes’, *Journal of British Studies*, 2018, 57, 755–59; Tara Mayer, ‘One British Thing: Jodhpurs’, *Journal of British Studies*, 2019, 58, 394–98. See also: Stephanie Downes, Sally Holloway and Sarah Randles, *Feeling Things: Objects and Emotions through History* (Oxford: Oxford University Press, 2018).

13 Stephen Hinchliffe *et al.*, ‘Healthy Publics: Enabling Cultures and Environments for Health’, *Palgrave Communications*, 2018, 57, 4, 6.

14 *Ibid.*, 7.

15 Andrew Seaton, ‘From Caution to Celebration: The NHS at 70’, *History of Government blog*, 5 July 2018 <https://history.blog.gov.uk/2018/07/05/from-caution-to-celebration-the-nhs-at-70/> (accessed 6 March 2019).

16 ‘The NHS: A People’s History’, *BBC Four website* <https://www.bbc.co.uk/programmes/p05rmvmh> (accessed 11 March 2019).

17 See videos at: ‘The People’s History of the NHS on the BBC’, *People’s History of the NHS website*, <https://peopleshistorynhs.org/#fndtn-videos> (accessed 11 March 2019).

18 ‘The Bellringer Babes’, *The NHS: A People’s History*, <https://www.bbc.co.uk/programmes/p06dxdwb/player> (accessed 14 August 2019).

19 On the cultural appeal and significance of television, see, for example: Joe Moran, *Armchair Nation: An Intimate History of Britain in front of the TV* (London: Profile Books, 2013).

20 ‘NHS on Film’, <https://player.bfi.org.uk/free/collection/nhs-on-film> (accessed 6 December 2019).

21 The Lothian Health Services Archive and King’s Fund Library and Information Services were critical in this research, uncovering and curating fascinating and relevant materials. Their websites and digitised collections may be accessed at, Lothian Health Services Archive <http://www.lhsa.lib.ed.ac.uk/> and King’s Fund Digital Archive <https://archive.kingsfund.org.uk/> (accessed 13 March 2019).

22 Victoria Bates, ‘Sensing Space and Making Place: The Hospital and Therapeutic Landscapes in Two Cancer Narratives’, *Medical Humanities*, 2019, 45, 10–20; Claire Hickman, *Therapeutic Landscapes: A History of English Hospital Gardens since 1800* (Manchester: Manchester University Press, 2013); Claire Hickman, ‘Cheerful Prospects and Tranquil Restoration: The Visual Experience of Landscape as part of the Therapeutic Regime of the British Asylum, 1800–1860’, *History of Psychiatry*, 2009, 20, 425–41. This trend was also represented at the European Association for History of Medicine and Health 2019 Biennal Conference, which included papers, for example, by Marie Alitt on the ‘sensory experiences of ventilation, air flow, and windows in the modern hospital’; by Victoria Bates on ‘defining noise and silence in the NHS hospital’; Michael Flexer on the waiting room; and Joana Balsa de Pinho on ‘architecture and the material culture in the renascence hospitals in Portugal’. See programme at: ‘2019 Conference Programme’, *European Association for the History of Medicine and Health*, <https://www.birmingham.ac.uk/Documents/college-mds/applied-health/SSiM/11.06.19-Final-Programme-for-EAHMH.pdf> (accessed 12 August 2019).

23 See short clip: <https://twitter.com/SundayBrunchC4/status/1017378042874880002> (accessed 14 August 2019).

24 See critique made by Dan Healey in: ‘Anniversaries’, *German History*, 2014, 32, 81–82; King and Rivett, ‘Engaging People in Making History’.

25 Fearghal McGarry, ‘Anniversaries’, *German History*, 2014, 32, 87.

26 Jörg Arnold, ‘Anniversaries’, *German History*, 2014, 32, 86.

27 See, for example: Emily Robinson *et al.*, ‘Telling Stories about Post-war Britain: Popular Individualism and the “Crisis” of the 1970s’, *Twentieth Century British History*, 2017, 28, 268–304; Matthew Hilton, Chris Moores and Florence Sutcliffe-Braithwaite, ‘New Times Revisited: Britain in the 1980s’, *Contemporary British History*, 2017, 31, 145–65.

28 Grady, ‘Anniversaries’, *German History*, 2014, 32, 80.

29 Roberta Bivins, ‘The Appointed Day – Celebrated or Silent?’, *People’s History of the NHS website*, <https://peopleshistorynhs.org/the-appointed-day-celebrated-or-silent/> (accessed 11 March 2019); Nick Hayes, ‘Did We Really Want a National Health Service in Britain? Hospitals, Patients, and Public Opinions before 1948’, *English Historical Review*, 2012, 526, 625–61 ; Jennifer Crane, ‘The NHS at 70: Its Greatest Achievements and the Power of Individual Stories’, *British Medical Journal Opinion*, 18 April 2018, <https://blogs.bmj.com/bmj/2018/04/18/the-nhs-at-70-its-greatest-achievements-and-the-power-of-individual-stories/> (accessed 6 March 2019).

30 Jennifer Crane, ‘Whose Needs? Histories of “Need” in the National Health Service’, *British Medical Journal Opinion*, 28 June 2018, <https://blogs.bmj.com/bmj/2018/06/28/jennifer-crane-whose-needs-histories-of-need-in-the-national-health-service/> (accessed 6 March 2019).

31 Mathijs Schiffers, ‘Holy British Care Cow is Limping and Top-Heavy’, *Het Financieele Dagblad*, 30 June 2018 <https://fd.nl/weekend/1259659/heilige-britse-zorgkoe-is-mank-en-topzwaar#> (accessed 6 March 2019).

32 Dan Healey, ‘Anniversaries’, *German History*, 2014, 32, 94.

33 Grace Huxford and Richard Wallace, ‘Voices of the University: Anniversary Culture and Oral Histories of Higher Education’, *Oral History*, 2016, 45, 79–90.

34 Joanna Bornat discusses the roots of the reminiscence movement with older people. While emphasising that it should not necessarily be framed as ‘Therapeutic’, given a lack of clear clinical evidence, she argues that it may nonetheless have benefits in terms of, for example, enabling participants to lead their own learning; building relationships between staff and elderly people they work with; and enabling all involved to make new connections between the past and present: Joanna Bornat, ‘Oral History as a Social Movement: Reminiscence and Older People’, *Oral History*, 1989, 17, 16–24. Jenny Harding discusses the idea of using oral history to ‘Give Voice’ to young care leavers, arguing that the theme of ‘Being Heard’, key to the *Care Stories* project, ‘Struck a Clear Chord with all Interviewees’: Jenny Harding, ‘Talk About Care: Emotions, Culture and Oral History’, *Oral History*, 2010, 38, 35, 39–40.

35 Jane Hand, ‘Childhood Vaccination and the NHS’, *People’s History of the NHS website*, <https://peopleshistorynhs.org/encyclopaedia/childhood-vaccination-and-the-nhs/> (accessed 11 March 2019); Jane Hand, ‘Premature Birth and the NHS’, *People’s History of the NHS website*, <https://peopleshistorynhs.org/encyclopaedia/premature-birth-and-the-nhs/> (accessed 11 March 2019).

36 Jennifer Crane, *Child Protection in England, 1960–2000: Expertise, Experience, and Emotion* (London: Palgrave Macmillan, 2018).

37 *Ibid*., 7; Carolyn Steedman, *Dust: The Archive and Cultural History* (Manchester: Manchester University Press, 2001).

38 In terms of gender, a relevant article is: Estella Tincknell, ‘The Nation’s Matron: Hattie Jacques and British Postwar Popular Culture’, *Journal of British Cinema and Television*, 2015, 12, 6–24. Roberta Bivins has also written an important article about how race was pictured in cultural depictions of the NHS within its first 40 years: Roberta Bivins, ‘Picturing Race in the British National Health Service, 1948–1988’, *Twentieth Century British History*, 2017, 28, 83–109.

39 Nicholas Stargardt, ‘Drawing the Holocaust in 1945’, *Holocaust Studies*, 2015, 11, 29, 31, 34.

40 See, for example: Sian Pooley and Colin Pooley, ‘“Such a Splendid Tale”: The Late Nineteenth-Century World of a Young Female Reader’, *Cultural and Social History*, 2005, 2, 329–51; Sian Pooley, ‘Children’s Writing and the Popular Press in England 1876–1914’, *History Workshop Journal*, 2015, 80, 75–98; Laura Tisdall, ‘“That was what Life in Bridgeburn had Made Her”: Reading the Autobiographies of Children in Institutional Care in England, 1918–46’, *Twentieth Century British History*, 2013, 24, 351–75; Catherine Sloan, ‘“Periodicals of an objectionable character”: Peers and Periodicals at Croydon Friends’ School, 1826–1875’, *Victorian Periodicals Review*, 2017, 50, 769–86; Hannah Elizabeth, 'Getting around the rules of sex education', *Wellcome Collection,* 7 June 2018 < https://wellcomecollection.org/articles/WxZnZyQAAPoF1PS8 > (accessed 22 April 2020); Sarah Kenny, ‘“Basically You Were Either a Mainstream Sort of Person or You Went to the Leadmill and the Limit”: Understanding Post-War British Youth Culture Through Oral History’, in Kristine Moruzi, Nell Musgrove and Carla Pascoe Leahy, eds, *Children’s Voices from the Past: New Historical and Interdisciplinary Perspectives* (London: Palgrave, 2019), 233–59.

41 Pooley, ‘Children’s Writing and the Popular Press in England’, 75.

42 Roberta Bivins, Hilary Marland and Nancy Tomes, ‘Histories of Medicine in the Household: Recovering practice and “Reception”’, *Social History of Medicine*, 2016, 29, 669–75. This article provides an introduction to a special section of *Social History of Medicine* discussing medicine in the household, including further contributions by Sandra Cavallo, Claire L. Jones, Tinde van Andel, and Marion Baschin.

43 Mathew Thomson, ‘The NHS and the Public: A Historical Perspective’, *King’s Fund*, 18 October 2017 <https://www.kingsfund.org.uk/blog/2017/10/nhs-and-public-historical-perspective> (accessed 11 March 2019).

44 A description of this research in relation to generation is forthcoming in: Jennifer Crane, ‘“Loving” the NHS: Social Surveys and Activist Feelings’, in Jennifer Crane and Jane Hand, eds, *Posters, Protests, and Prescriptions: Cultural Histories of the National Health Service in Britain* (under contract and forthcoming with Manchester University Press). To read more on this as a finding in contemporary social surveys, please see: Bobby Duffy *et al.*, *Generation Strains: A Demos and Ipsos MORI Report on Changing Attitudes to Welfare* (London: Demos, 2013).

45 Paul Long and Nick Webber, ‘“ … and Then There was one”’: Cultural Representations of the Last British Veteran of the Great War’, *Journal of War and Culture Studies*, 2019, 12, 139–55.

46 See, for example: Karen Rushton, ‘The Records of Stannington Children’s Sanatorium: Charting Half a Century of Tuberculosis Care’, *Social History of Medicine*, 2016, 29, 829–39. Emphasis on institutions ‘as objects of research’ rather than on the ‘domiciliary and informal provision of health care’ was also observed by Margaret Pelling in 1988 in ‘Child Health as Social Value in Early Modern England’, *Social History of Medicine*, 1988, 1, 137.

47 To take a few examples, historiographies of childhood disease often make significant—and important—use of records such as infant mortality and disease data, as well as clinical accounts of illness: Paul Atkinson *et al.*, ‘Patterns of Infant Mortality in Rural England and Wales, 1850–1910’, *Economic History Review*, 2017, 70, 1268–90; Emm Barnes Johnstone and Joanna Baines, *The Changing Faces of Childhood Cancer: Clinical and Cultural Visions since 1940* (Basingstoke: Palgrave Macmillan, 2015); Rushton, ‘The Records of Stannington Children’s Sanatorium, 829–39’; Kathleen Vongsathorn, ‘“A Real Home”: Children, Family, Mission, and the Negotiation of Life at the Kumi Children’s Leper Home in Colonial Uganda’, *Journal of the History of Childhood and Youth*, 2015, 8, 55–74. Significant historiographies also examine childhood disability: see on this the special issue on ‘Imperfect Children’ edited by Steven King and Steven J. Taylor for *Social History of Medicine*, 2017, 30, 718–26 . Notably, emergent historical accounts seek to look at childhood disease as a part of everyday life—and an often fleeting phenomenon, despite the challenges in accessing this, by using such diverse sources as recipe books and parental letters. See, for example: Hannah Newton, *The Sick Child in Early Modern England, 1580–1720* (Oxford: Oxford University Press, 2014); Edith Snook, ‘“The Women Know”: Children’s Diseases, Recipes and Women’s Knowledge in Early Modern Medical Publications’, *Social History of Medicine*, 2017, 30, 1–21.

48 Hannah Newton, ‘The Sick Child in Early Modern England, 1580–1720’, *Endeavour*, 2014, 38, 126–27.

49 Antoinette Burton, ed., *Archive Stories: Facts, Fictions, and the Writing of History* (Durham, North Carolina: Duke University Press, 2006); R. G. S. Carter, ‘Of Things Said and Unsaid: Power, Archival Silences, and Power in Silence’, *Archivaria: The Journal of the Association of Canadian Archivists*, 2006, 61, 215–33 ; Carolyn Steedman, *Dust: The Archive and Cultural History* (New Brunswick, NJ: Rutgers University Press, 2002); David Thomas, Simon Fowler and Valerie Johnson, *The Silence of the Archive* (London: Facet, 2017).

50 The following articles are powerful recent demonstrations of how oral history work changes the historical record, bringing new voices and memories into conversation with those which have already been documented: Pippa Virdee, ‘Remembering Partition: Women, Oral Histories and Partition of 1947’, *Oral History*, 2013, 41, 49–62; Jenny Harding, ‘Talk About Care: Emotions, Culture and Oral History’, *Oral History*, 34–42.

51 Paul Thompson’s classic article on oral history is useful in this regard, in terms of breaking down distinctions between ‘objective’ archival work and the ‘subjective’ work of listening to people’s voices: Paul Thompson, ‘Problems of Method in Oral History’, *Oral History*, 1972, 1, 1–47.

52 Hinchliffe *et al.*, ‘Healthy Publics’, 4.

53 Bivins, ‘The Appointed Day – Celebrated or Silent?’ ; Hayes, ‘Did we Really want a National Health Service in Britain?’ , 625–61.

54 Nick Mahony and Hilde C. Stephansen, ‘Engaging with the Public in Public Engagement with Research’, *Research for All*, 2017, 1, 35–71.

55 Anna Woodham *et al.* have discussed the creation of such ‘family archives’, which are created by ‘historians’ and ‘publics’, and indeed challenge these separated categories: Anna Woodham *et al.*, ‘We Are What We Keep: The “Family Archive”, Identity, and Public / Private Heritage’, *Heritage & Society*, Published Online 4 March 2019.

56 Katy Canales, ‘NHS Glasses’, *People’s History of the NHS website* <https://peopleshistorynhs.org/encyclopaedia/nhs-glasses/> (accessed 14 August 2019); Joanna Gooding, ‘Rather Unspectacular: Design Choices in National Health Service Glasses’, *Science Museum Group Journal*, Spring 2017.

57 Jane Hand, ‘Bow Arrow Hospital, 1953’, *People’s History NHS website* <https://peopleshistorynhs.org/the-childrens-ward-at-bow-arrow-hospital-1953/> (accessed 6 March 2019).

58 Alex Mold, ‘Repositioning the Patient: Patient Organizations, Consumerism and Autonomy in Britain During the 1960s and 1970s’, *Bulletin of the History of Medicine*, 2013, 87, 225–49.

59 Deborah Cohen, *Family Secrets: The Things We Tried to Hide* (London: Penguin, 2014).

60 Miri Rubin, ‘Presentism’s Useful Anachronisms’, *Past & Present*, 2017, 1, 236–44.

61 Alexandra Walsham, ‘Introduction: Past and … Presentism’, *Past & Present*, 2017, 234, 213–17.

62 Ann Oakley, ‘Interviewing Women: A Contradiction in Terms?’, in H. Roberts, ed., *Doing Feminist Research* (London: Routledge and Kegan Paul, 1981), 30–61; Ann Oakley, ‘Interviewing Women Again: Power, Time and the Gift’, *Sociology*, 2015, 50, 195–213 . Joanna Bornat also discusses how, in the early days of oral history in the early seventies, ‘it was the interviewee who reminded the historian that this was a shared experience’ (Bornat, ‘Oral History as a Social Movement’, 17.)

63 Janet Fink, ‘Foreword’, in Tracey Loughran and Dawn Mannay, eds, *Emotion and the Researcher: Sites, Subjectivities, and Relationships* (Bingley: Emerald Publishing Limited, 2018), xvii.

64 *Ibid*.

65 Wendy Rickard, ‘Oral History – “More Dangerous Than Therapy”?’ Interviewees’ Reflections on Recording Traumatic or Taboo Issues’, *Oral History*, 1998, 26, 40–41.

66 Mary Morris and Andrea Davies, ‘Being Both Researcher and Subject: Attending to Emotion within Collaborative Inquiry’, in Loughran and Mannay, *Emotion and the Researcher*, 229–44.

67 Heather Savigny, ‘The Violence of Impact: Unpacking Relations Between Gender, Media and Politics’, *Political Studies Review*, online access 26 February 2019, https://doi.org/10.1177/1478929918819212.

68 Forthcoming full description of these results in: Crane, ‘“Loving” the NHS: Social Surveys and Activist Feelings’, in Crane and Hand, eds, *Posters, Protests, and Prescriptions* .

69 See, for example: Wellcome Library, SA / PHG 3, Politics of Health Group Publications, Cuts and the NHS: Pamphlet Number Two, Politics of Health Group, 2.

70 Lucy Delap, ‘Feminism, Masculinities and Emotional Politics in Late Twentieth Century Britain’, *Cultural and Social History*, 2018, 15, 580.

71 Sarah Crook and Signy Gutnick Allen, ‘In Conversation with the Women’s Liberation Movement: Intergenerational Histories of Second Wave Feminism, British Library, 12 Oct. 2013’, *History Workshop Journal*, 2014, 77, 341, 342.

72 Delap, ‘Feminism, Masculinities and Emotional Politics’, 583.

73 *Ibid*., 588.

74 Crook and Gutnick Allen, ‘In Conversation with the Women’s Liberation Movement’, 340.

75 Chris Millard, ‘Using Personal Experience in the Academic Medical Humanities: A Genealogy’, *Social Theory & Health*, Advance access online 13 February 2019, https://doi.org/10.1057/s41285-019-00089-x.

76 Quote is from Sharlene Nagy Hesse-Bibber and Deborah Piatelli, ‘The Feminist Practice of Holistic Reflexivity’, in Sharlene Nagy Hesse-Biber, ed., *Handbook of Feminist Research: Theory and Praxis* (2nd edition, Thousand Oaks: SAGE, 2012), Chapter 27, 'The Feminist Practice of Holistic Reflexivity', 6. For further discussion of these ideas, see, for example, Dorothy E. Smith’s discussion of how women do not ‘enter and occupy’ sociology ‘on the same terms as men enter and occupy it’: Dorothy E. Smith, ‘Women’s Perspective as a Radical Critique of Sociology’, *Sociological Inquiry*, 1974, 44, 10..

77 Woodham *et al.*, ‘We Are What We Keep’, 203–20 .

78 The idea of a ‘public-expert gap’ is explored in: Anthony Giddens, *The Consequences of Modernity* (California: Stanford University Press, 1990); Frank Fischer, *Democracy and Expertise: Reorienting Policy Inquiry* (Oxford: Oxford University Press, 2009).

79 Ellen Stewart, *Publics and Their Health Systems: Rethinking Participation* (Basingstoke: Palgrave Macmillan, 2016).

80 Alix Green, *History, Policy and Public Purpose: Historians and Historical Thinking in Government* (Basingstoke: Palgrave Macmillan, 2016).

81 See: ‘Impact: How John Lewis Helped us Prove the Business Value of Archives’, *University of Essex website*, <https://www.essex.ac.uk/research/showcase/john-lewis-archives> (accessed 5 December 2019); Alix R. Green, ‘“Secret Lists and Sanctions”: The Blacklisting of the John Lewis Partnership and the Politics of Pay in 1970s Britain’, *Twentieth Century British History*, 2019, 30, 205–30. See also Working Knowledge project website, <http://www.workingknowledgeps.com/?page_id=223> (accessed 13 March 2019).

82 See: Loughran and Mannay, eds, *Emotion and the ResearcheNr*; Oakley, *Fracture*; Harding, ‘Talk About Care’, 33–42 ; Sophie Rees, ‘An Exploration of the Experiences and Perceptions of Young Women Living with a History of Breast Cancer in the UK’ (unpublished PhD thesis, University of Warwick, 2015), <https://ethos.bl.uk/OrderDetails.do?uin=uk.bl.ethos.682911> (accessed 21 November 2019), chapters 1 and 2; Hesse-Bibber and Piatelli, ‘The Feminist Practice of Holistic Reflexivity’, Chapter 27 ; Smith, ‘Women’s Perspective as a Radical Critique of Sociology, 7–13’.

83 Felicity Callard and Des Fitzgerald, *Rethinking Interdisciplinarity Across the Social Sciences and Neurosciences* (Basingstoke: Palgrave Macmillan, 2015).

84 See on this: Tom Sperlinger, Josie McLellan and Richard Pettigrew, *Who are Universities For? Remaking Higher Education* (Bristol: Bristol University Press, 2018).

9 Jennifer Crane, ‘Visit to Leamington Spa History Society’, *People’s History of the NHS website*, <https://peopleshistorynhs.org/newsandevents/visit-to-leamington-spa-history-society/> (5 March 2019).

## Notes

Jennifer Crane is a historian at the University of Oxford, currently leading a Wellcome Trust-funded research project about the experiences and mobilisation of gifted children in the post-war world. From 2016 to 2019, she was a Public Engagement Research Fellow on the Cultural History of the NHS project at the University of Warwick, which was funded by a Wellcome Trust Senior Investigator Award.

